# Darkness and Light

**Published:** 1864-01

**Authors:** 


### DARKNESS AND LIGHT.

THERE is no heart but hath its inner anguish,
  There is no eye but hath with tears been wet;
There is no voice but hath been heard to languish
  O'er hours of darkness it can ne'er forget.

There is no cheek, however bright its roses,
  But perished buds beneath its hues are hid;
No eye that in its dewy light reposes,
  But broken star-beams tremble 'neath its lid.

There is no lip, howe'er with laughter ringing,
  However bright and gay its words may be,
But it hath trembled at some dark upspringing
  Of stern affliction and deep misery.

We all are brothers in this land of dreaming,
  Yet hand meets hand, and eye to eye replies;
Nor dream we that beneath an eye all beaming
  The flower of life in broken beauty lies.

O blessed light, that gilds our night of sorrow!
  O balm of Gilead, for our healing found!
We know that peace will come with thee to-morrow,
  And that afflictions spring not from the ground.

